# Intestine-Targeted DGAT1 Inhibition Improves Obesity and Insulin Resistance without Skin Aberrations in Mice

**DOI:** 10.1371/journal.pone.0112027

**Published:** 2014-11-18

**Authors:** Naoto Tsuda, Shin Kumadaki, Chika Higashi, Makoto Ozawa, Mikihiko Shinozaki, Yutaka Kato, Koutarou Hoshida, Satomi Kikuchi, Yoshihisa Nakano, Yoshihiro Ogawa, Shoji Furusako

**Affiliations:** 1 Discovery Research, Mochida Pharmaceutical Company Limited, Shizuoka, Japan; 2 Department of Molecular Endocrinology and Metabolism, Graduate School of Medical and Dental Sciences, Tokyo Medical and Dental University, Tokyo, Japan; Northeast Ohio Medical University, United States of America

## Abstract

**Objective:**

Diacylglycerol O-acyltransferase 1 (DGAT1) catalyzes the final committed step in triglyceride biosynthesis. DGAT1 null mice are known to be resistant to diet-induced obesity, and more insulin sensitive relative to the wild-type; however, the mice exhibit abnormalities in the skin. This work determined whether the intestine-targeted DGAT1 inhibitor could improve obesity and insulin resistance without skin aberrations in mice.

**Design and Methods:**

We synthesized 2 DGAT1 inhibitors: Compound A, described in the patent application from the Japan Tobacco, and Compound B (A-922500), reported by Abbott Laboratories. Both compounds were evaluated for inhibitory activities against DGAT1 enzymes and effects on the skin in mice *in vivo.* Compound B was further investigated for effects on obesity and insulin resistance in diet-induced-obese (DIO) mice.

**Results:**

The 2 compounds comparably inhibited the DGAT1 enzyme activity and the cellular triglyceride synthesis *in vitro*, while they showed different distribution patterns in mice *in vivo*. Compound A, which distributed systemically, caused skin aberrations, while Compound B, which preferentially distributed to the intestine, improved obesity and insulin resistance without skin aberrations in DIO mice.

**Conclusions:**

Our results suggest that the intestine is the key tissue in which DGAT1 plays a role in promoting obesity and insulin resistance.

## Introduction

Diacylglycerol O-acyltransferase (DGAT) 1 and DGAT2 catalyze the final committed step in triglyceride biosynthesis [1]. DGAT1 null mice are resistant to diet-induced obesity, and exhibit higher insulin sensitivity compared to the wild-type [2]–[4]. These results have revealed that DGAT1 plays important roles in the triglyceride synthesis and regulation of energy metabolism. Similarly, DGAT2 also plays a role in mammalian triglyceride synthesis. Although plasma glucose, triglyceride, free fatty acid levels and liver glycogen are decreased in DGAT2 null mice, these mice are lipopenic and die soon after birth due to reduction in substrates for energy metabolism and profound skin aberrations [5]. Thus, the DGAT1 inhibitor is expected to become a novel treatment option for obesity and type-2 diabetes mellitus (T2DM) without the severe adverse events associated with the DGAT2 inhibition. However, DGAT1 null mice exhibit skin aberrations, presumably due to depletion of essential lipids within the skin [3], and thus the DGAT1 inhibitor could induce skin aberrations when systemically administered for the treatment of obesity and diabetes.

Orally taken triglycerides go through hydrolysis and then are rearranged within the enterocyte into triglyceride-rich chylomicrons destined for systemic circulation. Since DGAT1 catalyzes this triglyceride rearrangement, the improvement of metabolic disorders observed in DGAT1 null mice seems to be derived from lack of DGAT1 activity in the intestine. Moreover, the metabolic phenotypes of DGAT1 null mice such as resistance to hepatic steatosis and diet-induced obesity are reported to be lost when DGAT1 is reintroduced into the intestine [6]. Thus the inhibition of the intestinal DGAT1 may be sufficient to improve metabolic disorders, suggesting that the intestine-targeted DGAT1 inhibitor shows beneficial metabolic effects without skin aberrations. Indeed, a previous report [7] aimed for discovery and optimization of intestine-targeted DGAT1 inhibitors. However, detailed comparisons between systemic delivery and intestine-targeted delivery of DGAT1 inhibitors have not been made so far.

In this study, we evaluated the effects of 2 DGAT1 inhibitors with different distribution patterns on skin aberrations in mice. As a result, we have found that the predominantly intestine-targeted DGAT1 inhibitor attenuates metabolic disorders without inducing skin aberrations.

## Materials and Methods

### Compounds

**Figure 1:**
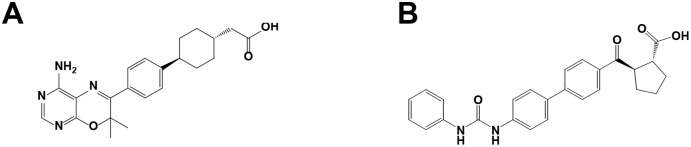
Chemical structures of Compound A (A) and Compound B (B).

Compound A (4-[4-(4-amino-2,7,7-trimethyl-7H-pyrimido[4,5-b][1], [4]oxazin-6-yl)phenyl]-trans-Cyclohexaneacetic acid, Figure 1A), described in the patent application from the Japan Tobacco [8], has been reported as a potent DGAT1 inhibitor by AstraZeneca [9]. Compound B (A-922500, (1R, 2R)-2-[[4′-[[(Phenylamino)carbonyl]amino][1,1′-biphenyl]-4-yl]carbonyl]cyclopentanecarboxylic acid, Figure 1B) has also been reported as a potent DGAT1 inhibitor by Pfizer and Abbott Laboratories [10], [11]. Compound A and B act on the acyl-CoA binding site of DGAT1 and that they compete each other for the binding to DGAT1 [10]. These compounds were synthesized in our laboratory and used for the present study.

### Animals

Male C57BL/6J mice were purchased from Charles River Japan. Mice were fed ad libitum with either a standard rodent chow (CE-2; CLEA Japan, Tokyo, Japan) or a high-fat diet (D12492; Research Diets, New Brunswick, NJ). All animals were housed under a 12-h light-dark cycle (lights on from 7∶30 am to 7∶30 pm) with free access to food and tap water.

All experimental procedures were approved by the Institutional Animal Care and Use Committee of Mochida Pharmaceutical Research Center. Blood sample was withdrawn from venous blood vessels using heparinized syringe. All surgery was performed under isoflurane anesthesia, and all efforts were made to minimize suffering.

### Enzyme assays

The effects of the DGAT1 inhibitors were assessed using human (BC015762 from HeLa cell) and mouse DGAT1 (NM_010046 from mouse testis) in Sf9 insect microsomes. To collect the cell S9 fraction, sf9 cells were infected with the recombinant baculovirus containing human DGAT1 coding sequences and harvested after 72 h. The Sf9 cells were homogenized and centrifuged at 600 g for 20 min. Thereafter, the supernatant was centrifuged at 10000 g for 1 h and the precipitate was collected as the S9 fraction.

The reaction was started by adding ^14^C decanoyl CoA (0.85 kBq; American Radiolabeled Chemicals Inc., Saint Louis, MO) to 5 µg/ml protein of the S9 fraction with Compound A or B, 150 mM MgCl_2_, 0.01% BSA and 0.15 mM dioleoyl-glycerol in a total volume of 100 µl in 96-well plates. The mixture was incubated at room temperature for 45 min. The reaction was stopped by adding 0.5 M HCl and the incubated mixture was filtered using Multiscreen_HTS_-FB (Millipore, Billerica, MA). The human DGAT1 activity was quantified by counting the radioactivity of synthesized ^14^C triglycerides by the Microbeta scintillation counter (PerkinElmer, Waltham, MA) in duplicate. The 50% inhibitory concentration (IC_50_) was calculated using the sigmoidal curve-fitting program (Symyx Assay Explorer 3.1 SP2; Symyx Technologies Inc., Santa Clara, CA). Mouse DGAT1 enzyme assays were carried out in the same way.

### Triglyceride synthesis in HT-29 cells and HepG2 cells

The HT-29 cell line (American Type Culture Collection, Manassas, VA) was maintained in the McCoy’s 5A medium (Life Technologies, Carlsbad, CA) containing 10% fetal bovine serum. The HepG2 cell line (American Type Culture Collection) was maintained in the Dulbecco’s Modified Eagle Medium (Sigma-Aldrich, St. Louis, MO) containing 10% fetal bovine serum. For the experiments, the HT-29 cells were plated in 12-well plates at 3×10^5^ cells, the HepG2 cells were plated in 12-well plates at 5×10^5^ cells. The cells were cultured in serum-free media for 24 h, and incubated with a compound and oleic acid-albumin (Sigma-Aldrich) solubilized in dimethyl sulfoxide (final 0.1%) for 30 min. The triglyceride synthesis was initiated by the addition of ^14^C oleic acid (0.2 µCi). The cells were washed in PBS and harvested into the organic phase using hexane:2-propanol (3∶2) 30 min after initiation of the triglyceride synthesis. The solvent was evaporated, the extracts were solubilized in chloroform, and lipids were separated via TLC. Incorporation of the radiolabel into the triglyceride fraction was analyzed using BAS2000 in triplicate. The IC_50_ of each compound was calculated using GraphPad Prism.

### Compound distribution in mice

Exposure was analyzed using blood, skin, and intestine samples from the C57BL/6J mice orally treated with 30 mg/kg of a compound. At 1, 4, 8, and 24 h after the administration, mice were anesthetized with isoflurane, and blood, skin, and intestine samples were collected. Three mice were used in each group. The blood samples were collected with sodium heparin, subjected to centrifugation, and plasma samples were collected. The skin and the intestine were homogenized and centrifuged to collect samples. The compound concentrations in each tissue sample with values equal to or greater than the lower limit of quantification (LLOQ; 0.005 µg/ml) were used for analyses (HPLC-MS/MS: Shiseido, Tokyo, Japan, Thermofisher Scientific, Waltham, MA).

### Effects of Compound A and B on the skin in diet-induced-obese (DIO) mice

After 10 weeks on high-fat diet, mice became obese (36±0.24 g). Mice showed resistance to diet-induced obesity were excluded from the experiment. Fourteen-week-old male DIO mice were orally administered the vehicle (10% Pluronic F-68 for Compound A, 1% Tween 80 for Compound B), 30 mg/kg Compound A once daily or 30 mg/kg Compound B twice daily. These dosing regimens were decided based on the ratios of concentration at 24 h after the compound administration (*C*_24_) in the intestine/the IC50 for mDGAT1 (3.7 for Compound A and 1.2 for Compound B; Table 1) so that both compounds inhibit the intestinal DGAT1 to the same extent at 24 h after their administration. After 4 weeks of the treatment, all the animals were sacrificed. The skin (dorsolateral lesion) was removed and then fixed in 10% neutral buffered formalin, processed, embedded in paraffin, cut into thin sections and stained with Hematoxylin and Eosin. Histopathological examinations were performed. The size of the sebaceous glands were measured using WinROOF (Mitani Corporation, Tokyo, Japan).

**Table 1:** Pharmacokinetic properties of Compound A and Compound B in mice.

	Compound A	Compound B
*T*_max_ (h)	1	1
*C*_max_ in plasma (µM)	240±59	9.1±2.3
plasma/IC_50_	38±9.2	0.59±0.15
*C*_max_ in skin (µmol/kg)	14±0.62	1.3±0.51
skin/IC_50_	2.2±0.097	0.084±0.033
*C*_max_ in intestine (µmol/kg)	77±37	92±36
intestine/IC_50_	12±5.8	5.9±2.3
*C*_24_ in intestine (µmol/kg)	24±1.6	19±3.7
*C*_24_ in intestine/mDGAT1 IC_50_	3.7±0.24	1.2±0.24
Intestine/Plasma ratio at *T*_max_	0.32±0.090	10±2.8
Intestine/Skin ratio at *T*_max_	5.6±2.5	74±8.4

plasma/IC_50_ = *C*_max_ in plasma/mDGAT1 IC_50_, skin/IC_50_ = *C*_max_ in skin/mDGAT1 IC_50_, intestine/IC_50_ = *C*_max_ in intestine/mDGAT1 IC_50_, Three mice were used in each group. Data were expressed as means ± SD.

### Effects of Compound B on postprandial hyperlipidemia in mice

Eight-week-old male C57BL/6J mice were divided into treatment groups, each consisting of 7–8 mice, based on the body weight for the purpose of simplification of dose calculation. Mice were orally administered 1% Tween 80 (vehicle), 0.3, 1, 3, 10, or 30 mg/kg Compound B. Twelve h after administration, mice were orally given 3 ml/kg corn oil. Blood samples were collected 1, 2, and 3 h later, and the plasma triglyceride level was determined by using WAKO TG H (Wako, Osaka, Japan). The area under the curve (AUC) of plasma triglyceride (AUCtriglyceride) was calculated.

### Effects of Compound B on glucagon-like peptide-1 (GLP-1) secretion in mice

Mice were divided into treatment groups, each consisting of 6–8 mice, based on the body weight for the purpose of simplification of dose calculation. Mice were orally administered 1% Tween 80 (vehicle), 10, or 30 mg/kg Compound B. Thirty minute after administration, mice were orally given 3 ml/kg corn oil. Blood samples were collected 2 h later, and the plasma GLP-1 level was determined by using Glucagon Like Peptide-1 (Active) ELISA Kit (Linco Research, Inc., St. Charles, MO).

### Long-time treatment with Compound B in DIO mice

For evaluation of efficacy of long-time treatment with Compound B, after 10 weeks on high-fat diet, 14-week-old male mice were divided into treatment groups, each consisting of 9–10 animals, based on the body weight and glucose levels. Mice were orally administered 1% Tween 80 (vehicle), 1, 3, 10 or 30 mg/kg Compound B twice daily for 4 weeks. The DIO mice were fed with high-fat diet during the 4-week treatment period. Mice were fasted for 4 hours prior to blood collection. Plasma glucose, insulin, triglyceride, and free fatty acid concentrations were determined by using Glucose CII Test Wako (Wako), ultra sensitive mouse ELISA (Morinaga, Yokohama, Japan), L type WAKO TG H, and HA test WAKO NEFA (Wako), respectively.

### Statistical analyses

Data were expressed as means ± SEM or means ± SD. Statistical analyses between the vehicle-treated group and each compound-treated group were performed with the Dunnett’s multiple comparison test, and those between the vehicle-treated group and the control group were performed by the t-test, using SAS version 9.1 (SAS Institute Inc, Cary, NC).

## Results

### Characteristics of the tested compounds *in vitro*

Compound A and B were potent DGAT1 inhibitors for mouse and human DGAT1 (Table 2). These results were consistent with those in the previous reports [9]–[11].

**Table 2:** Summary of the effects of Compound A and Compound B.

	Compound A	Compound B
mDGAT1 IC_50_ a	6.4 nM	15.5 nM
hDGAT1 IC_50_ a	2.6 nM	8.4 nM
Log D	<0.9	<0.9
m plasma protein binding	3.2% free	0.1% free

a The 50% inhibitory concentration (IC_50_) for each compound in the enzymes expressed in insect microsomes.

### Inhibition of triglyceride synthesis *in vitro*

**Figure 2:**
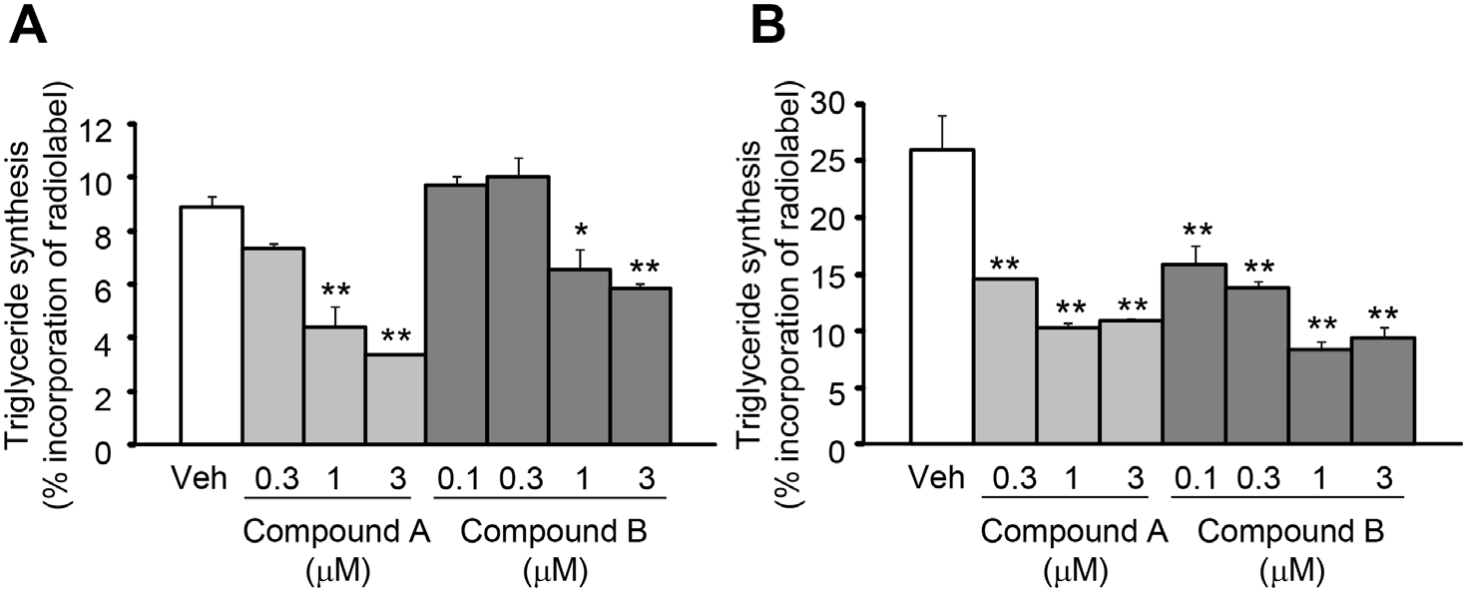
Effects of Compound A and Compound B on the triglyceride synthesis. The effects of Compound A and Compound B on the triglyceride synthesis in HT-29 human colon cells (A) and in HepG2 human liver cells (B). Data are expressed as means + SEM of 3 assays. *P<0.05 and **P<0.01. Statistical analyses between the vehicle (Veh) group and compound-treated groups were performed by the Dunnett’s multiple comparison test.

In the cellular assay, Compound A was evaluated at concentrations of 0.3 to 3 µM. Compound A inhibited the cellular triglyceride synthesis with the increase in concentrations, and significant inhibition was observed at 1 and 3 µM in HT-29 human colon (Figure 2A) and at all the concentrations tested in HepG2 human liver cells (Figure 2B). Compound B was evaluated at concentrations of 0.1 to 3 µM. Compound B also inhibited the cellular triglyceride synthesis with the increase in concentrations, and significant inhibition was observed at 1 and 3 µM in HT-29 human colon and at all the concentrations tested in HepG2 human liver cells.

### Distribution of DGAT1 inhibitors in mice

**Figure 3:**
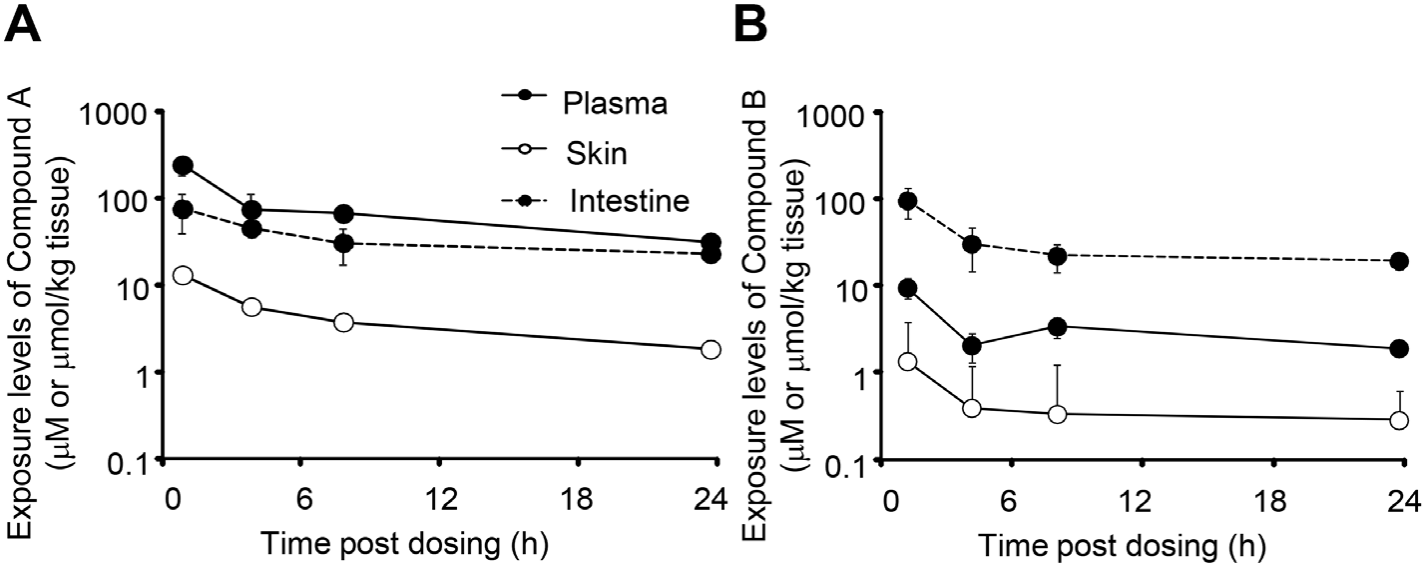
Plasma and tissue concentrations after single oral dosing of Compound A and Compound B. Plasma, skin, and intestine samples were collected from the mice orally administered 30 mg/kg Compound A (A) or B (B). Data are expressed as means ± SD of 3 animals.

Figure 3A and 3B show the concentrations of Compound A and B in the plasma, skin, and the intestine of the mice after its oral administration at 30 mg/kg. The time to reach the maximum concentration (Tmax) for Compound A was 1 h, and Compound A exhibited higher concentrations in the plasma and the skin as compared to Compound B (Table 1). The Tmax of Compound B was also 1 h. Compound B exhibited a higher concentration in the intestine in spite of its relatively low concentrations in the plasma and skin. The intestine/plasma and intestine/skin ratios for Compound B (10 and 74) were much higher than those for Compound A (0.32 and 5.6).

### Effects of DGAT1 inhibitors on the skin in diet-induced-obese (DIO) mice

**Figure 4:**
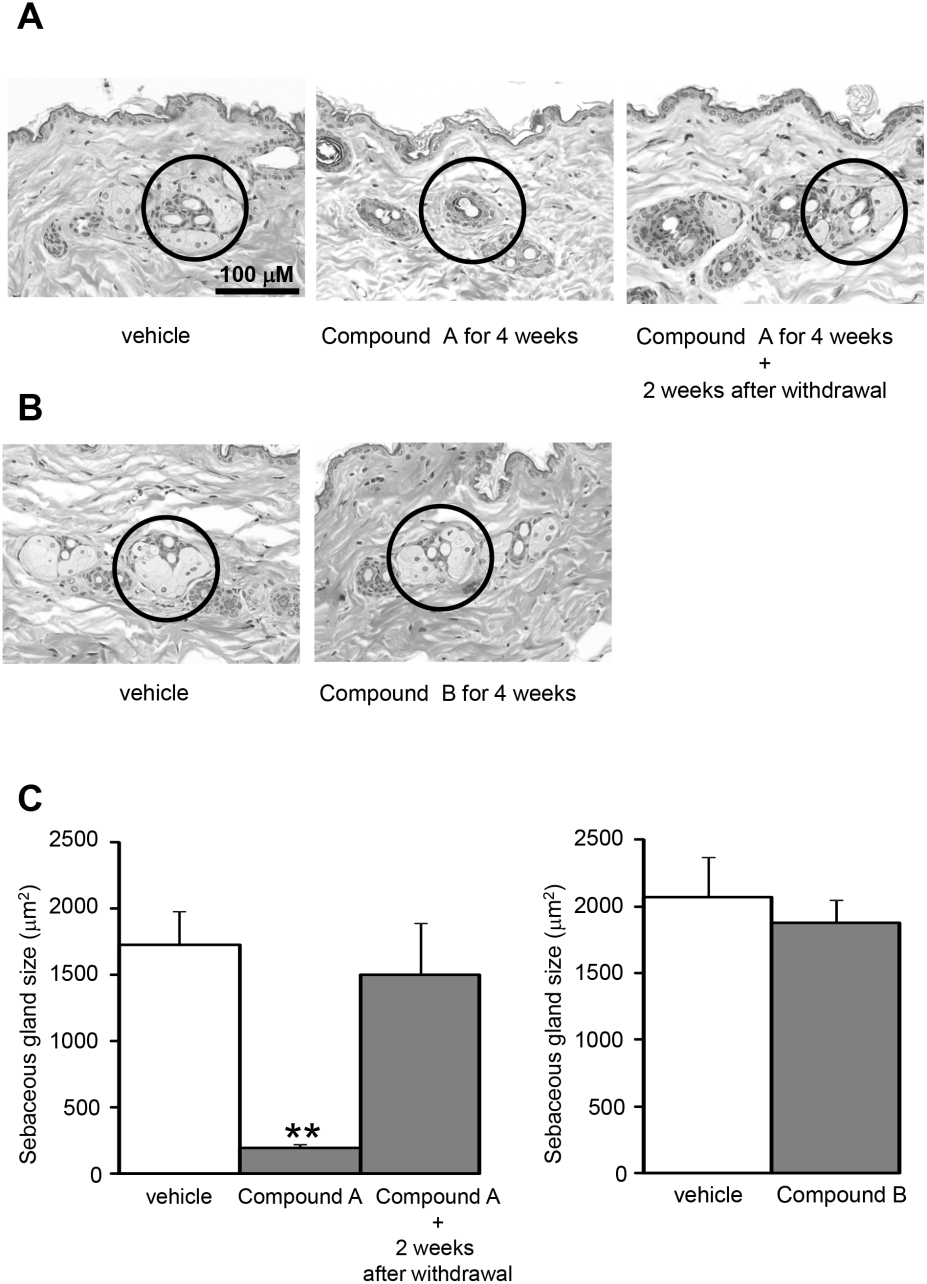
Microphotographs of the skin from the diet-induced-obese (DIO) mice. Microphotographs of the skin from the diet-induced-obese (DIO) mice treated with vehicle (10% Pluronic F-68 for Compound A, 1% Tween 80 for Compound B), 30 mg/kg Compound A once daily (A), or 30 mg/kg Compound B twice daily (B) for 4 weeks. The sebaceous gland is surrounded with a circle. The mice administered Compound A showed moderate skin aberrations derived from atrophy of the sebaceous gland. There were no injurious or inflammatory changes, and the skin aberrations recovered 2 weeks after the Compound A withdrawal. There were no skin aberrations in the mice administered Compound B. The sebaceous gland sizes are plotted (C). Data are expressed as means + SEM (N = 4). **P<0.01. Statistical analyses between the vehicle-treated group and Compound-treated groups were performed by the Dunnett’s multiple comparison test.

The DIO mice treated with Compound A showed skin aberrations derived from atrophy of the sebaceous gland which were characterized by decrease amount and size of sebaceous gland acini (Figure 4A and 4C). All the mice treated with Compound A exhibited skin aberrations, while such aberrations were not observed in the mice treated with the vehicle. Compound A induced no injurious or inflammatory changes, and the skin aberrations recovered 2 weeks after the compound withdrawal. On the other hand, the changes in the skin were not observed in all the DIO mice treated with Compound B, which displayed higher distribution to the intestine over plasma or the skin (Figure 4B and 4C).

### Effects of short-time treatment with the intestine-targeted DGAT1 inhibitor on postprandial hyperlipidemia in mice

**Figure 5:**
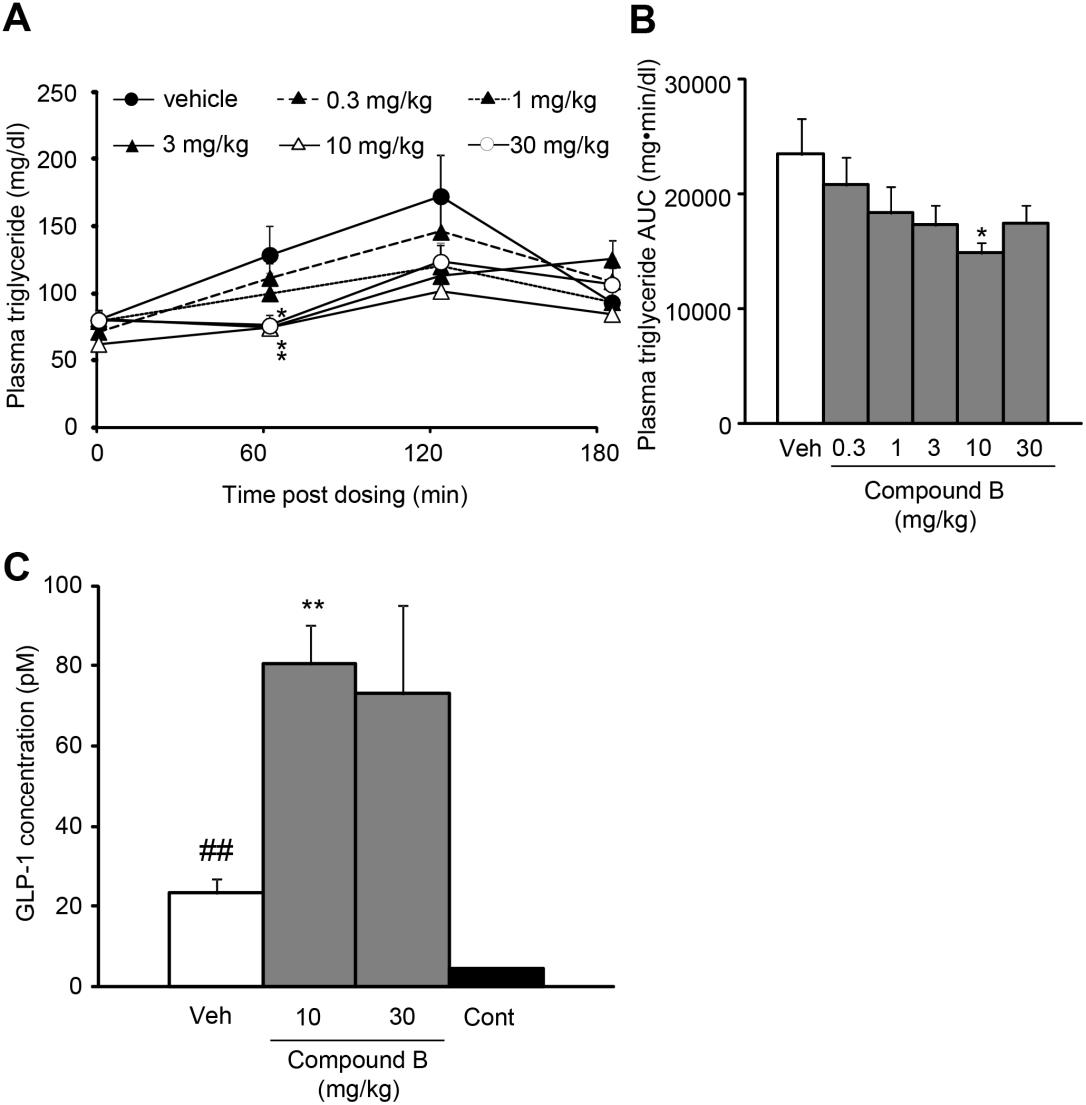
Effects of Compound B on the plasma triglyceride (A, B) and glucagon-like peptide-1 (C) levels. The changes in plasma triglyceride (A) and AUCtriglyceride (−5−180 min) of plasma triglyceride (B), and the plasma GLP-1 levels 2 h following corn oil administration (C) are indicated. The plasma GLP-1 levels of the mice given corn oil are higher than the control mice (Cont). Data are expressed as means + SEM of 6–8 animals. *P<0.05 and **P<0.01. Statistical analyses between the vehicle (Veh)-treated group and Compound B-treated groups were performed by the Dunnett’s multiple comparison test. ##*P*<0.01. Statistical analyses between the vehicle-treated group and the control group were performed by the t-test.

Compound B was evaluated in a model of postprandial hyperlipidemia by measuring chylomicron-derived triglycerides. Compound B at 0.3, 1, 3, or 10 mg/kg, when orally administered 12 h before the corn oil administration, reduced plasma triglyceride levels with a dose-dependent tendency (Figure 5). The maximum efficacy was achieved at 10 mg/kg. In addition, compound B at 10 and 30 mg/kg also increased plasma glucagon-like peptide-1 (GLP-1) levels after corn oil administration (Figure 5C).

### Effects of long-time treatment with the intestine-targeted DGAT1 inhibitor on diet induced obesity in mice

**Figure 6:**
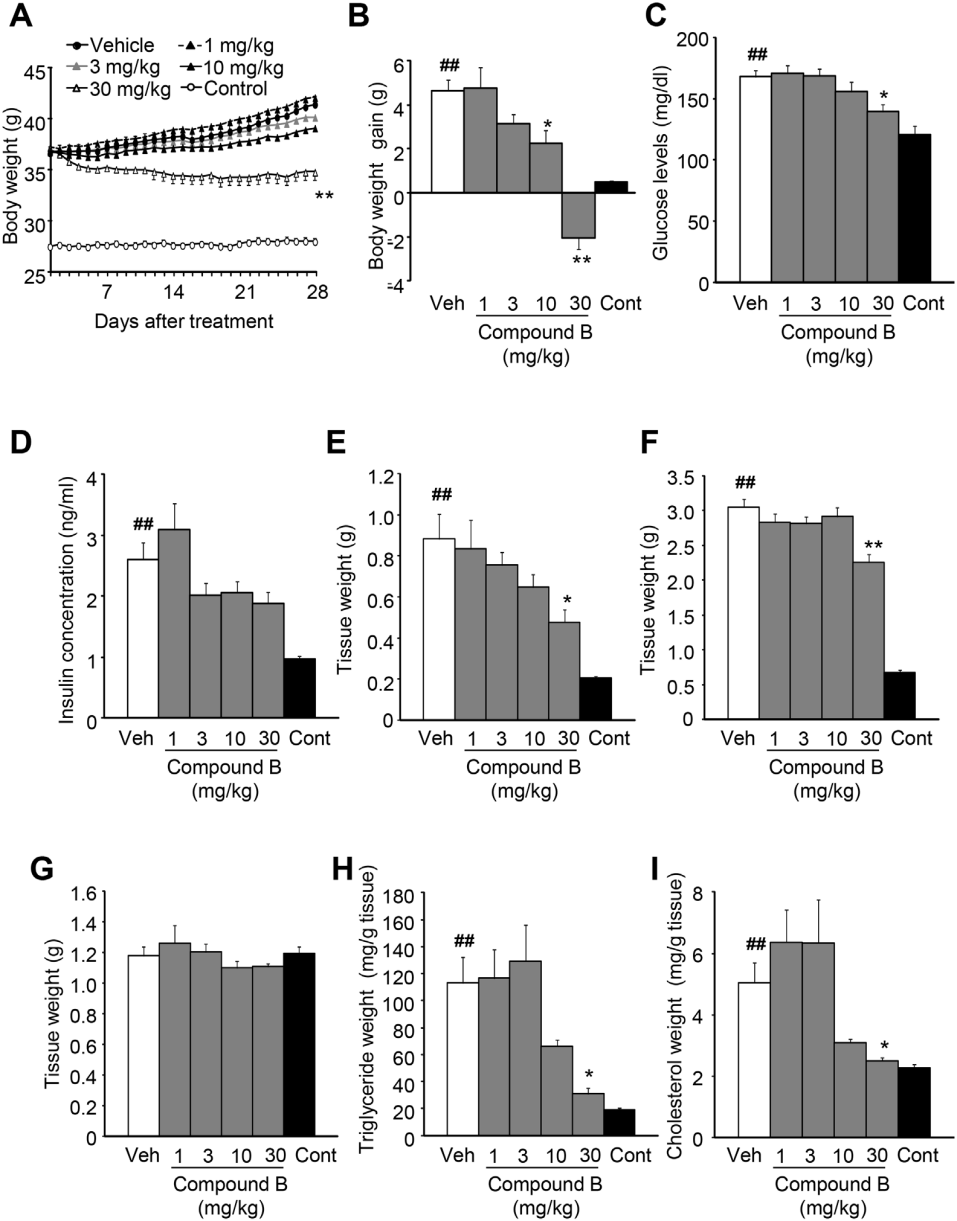
Effects of Compound B in diet-induced-obese (DIO) mice. Mice were orally treated with 1% Tween 80 (vehicle; Veh) or 30 mg/kg Compound B twice daily for 4 weeks. The time-course of body weight changes (A) and the body weight gain at 4 weeks of Compound B treatment (B) are indicated respectively. The body weight of the mice fed a high-fat diet is significantly higher than the control mice (Cont) fed a standard rodent chow during the 4-week treatment period. The plasma glucose level (C), plasma insulin concentration (D), mesenteric adipose tissue weight (E), epididymal adipose tissue weight (F), liver weight (G), content of hepatic triglyceride (H), and the content of hepatic total-cholesterol (I) at 4 weeks of Compound B treatment are indicated respectively. Data are expressed as means ± SEM of 9–10 animals. *P<0.05 and **P<0.01. Statistical analyses between the vehicle-treated group and Compound B-treated groups were performed by the Dunnett’s multiple comparison test. ##*P*<0.01. Statistical analyses between the vehicle-treated group and the control group were performed by the t-test.

As short-time treatment with Compound B improved postprandial hyperlipidemia, potential anti-obesity effects of Compound B were examined in DIO mice. Following the feeding on high-fat diet for 10 weeks, mice became obese, and exhibited significant increases in the body weight, plasma glucose level, plasma insulin concentration, insulin resistance (HOMA-IR: control vs. DIO = 0.70 vs. 2.0), mesenteric adipose tissue weight, epididymal adipose tissue weight, content of hepatic triglyceride and the content of hepatic total-cholesterol, indicating the development of diet-induced obesity in mice (i.e., DIO mice), as compared to the control mice fed on normal diet. In DIO mice, long-time treatment with Compound B attenuated the body weight gain with the increase in doses (Figure 6A). After the 4-week treatment period, significant attenuation and significant reduction were observed at 10 mg/kg and 30 mg/kg, respectively (Figure 6B). Compound B reduced plasma glucose levels (Figure 6C), and significant reduction was observed at 30 mg/kg. Insulin concentrations tended to decrease in the DIO mice treated with Compound B (Figure 6D). There were also differences in the weight of white adipose tissue (WAT) among the groups. Treatment with Compound B reduced the weight of mesenteric (Figure 6E) and epididymal (Figure 6F) fat with the increase in doses and significant reduction was observed at 30 mg/kg. Furthermore, despite lack of differences in the liver weight among the groups (Figure 6G), Compound B reduced concentrations of triglyceride and total-cholesterol in the liver with the increase of doses, and significant reduction was observed at 30 mg/kg (Figure 6H, 6I). Histological analysis also indicated that Compound B attenuated fatty liver (Figure S1).

## Discussion

DGAT1 is an endoplasmic membrane bound enzyme which catalyzes the final committed step in triglyceride biosynthesis, distributed in various tissues such as the skeletal muscle, liver, adipose tissue, intestine and the skin. In addition to the metabolic phenotypes of DGAT1 null mice such as resistance to hepatic steatosis and diet-induced obesity, they exhibit skin aberrations owing to lack of DGAT1 in the skin [3]. As the metabolic phenotypes of DGAT1 null mice are lost when DGAT1 is reintroduced into the intestine [6], we raised a hypothesis that the intestine-targeted DGAT1 inhibitor would improve metabolic disorders with minimizing the adverse effect that may be driven by systemic DGAT1 inhibition. In this study, we focused on effects of the 2 DGAT1 inhibitors with different distribution patterns on skin aberrations in mice. Consequently, we found that the predominantly intestine-targeted DGAT1 inhibitor Compound B did not exhibit skin aberrations, while the systemically distributed Compound A caused skin aberrations in DIO mice. We also indicated that the intestine-targeted DGAT1 inhibitor improved metabolic disorders in DIO mice.

The present result showed that Compound A and B similarly inhibited the activities of the mouse and the human DGAT1 enzyme *in vitro* as previously reported [9]–[11]. Moreover, both compounds inhibited the cellular triglyceride synthesis in HT-29, HepG2, and mouse skin homogenates. In spite of similar profiles *in vitro*, we identified that orally administered Compound A and B exhibited different distribution patterns in mice *in vivo*. The recent publication [12] indicates that distribution of DGAT1 inhibitors to the skin did correlate with its lipophilicity. However lipophilicity (Log D) of Compound A and B were similarly low. Although the precise reason for the difference of distribution has not yet been identified, the intestine-targeted distribution of Compound B may potentially be due to its transporter-mediated efflux from the intestine in the same way as previously discussed with another intestine-targeted DGAT1 inhibitor [7]. It is noteworthy that the intestine/plasma and intestine/skin ratios for Compound B (10 and 74) were much higher than those for Compound A (0.32 and 5.6). Since there is a report that Compound A shows beneficial metabolic effects [10], we focused on whether the predominantly intestine-targeted DGAT1 inhibitor could improve obesity and insulin resistance without skin aberrations in mice in the present study.

In a model of postprandial hyperlipidemia measuring chylomicron-derived triglycerides in mice, single oral administration of Compound B reduced plasma triglyceride levels. This is consistent with the previous observations on the suppression by other DGAT1 inhibitors of postprandial hyperlipidemia [9]–[11] and the delayed fat absorption in DGAT1 null mice [13]–[14]. Furthermore, Compound B also increased plasma GLP-1 levels after corn oil administration. The present results support the hypothesis that intestinal DGAT1 might directly regulate the releases of GLP-1 [15]. It might be possible that diacylglycerol accumulated in the intestinal cells following oil administration stimulates the release of GLP-1, since it has been known that the activation of phorbol ester-sensitive protein kinase C leads to GLP-1 release. These results highlighted that pharmacological as well as genetic inhibition of DGAT1 in the intestine would reduce dietary fat absorption and increase plasma GLP-1 levels.

Long-time treatment with Compound B, which selectively inhibits intestinal DGAT1, reduced the body weight gain, weight of white adipose tissues, hepatic triglyceride and the hepatic cholesterol content in DIO mice. In addition, long-time treatment with Compound B reduced plasma glucose levels and tended to reduce insulin concentrations, suggesting that Compound B would improve insulin resistance. In the recent publication [12], the pharmacokinetic/pharmacodynamic relationship of DGAT1 inhibitors between tissue concentration/IC_50_ and its effects was reported. High value (>1) of intestine/IC_50_ of Compound B in spite of its relatively low values of skin/IC_50_ and plasma/IC_50_ supports the consideration that the effects of DGAT1 inhibitors are achieved by the compound in intestine. The beneficial metabolic effects of Compound B may, at least partly, be ascribed to suppressed fat absorption from the intestine via inhibited intestinal DGAT1, as postprandial hyperlipidemia is assumed to promote hepatic steatosis [16] and insulin resistance. In addition to reduction of fat absorption, since it is known that GLP-1 has beneficial metabolic effects such as reduction of body weight gain and improvement of insulin resistance, increased GLP-1 secretion via the inhibition of intestinal DGAT1 would also contribute to the favorable metabolic effects of Compound B.

It is well known that obesity, particularly excessive triglyceride deposition in the non-adipose tissues such as the skeletal muscle and the liver, is related to diabetes and insulin resistance [17]–[21]. DGAT1 inhibition in the whole body may reduce excessive triglyceride deposition in the non-adipose tissues including the skeletal muscle [22], resulting in amelioration of metabolic disorders. In contrast, however, the inhibition of DGAT1 activities in the skeletal muscle or macrophage may cause insulin resistance, leading to aggravation of metabolic disorders, as a result of inhibited conversion of fatty acids substrates which induce insulin resistance into the form of triglyceride [23], [24]. Those opposing actions of DGAT1 may explain the reasons why the reintroduction of DGAT1 into the intestine of DGAT1 null mice is sufficient to lose metabolic phenotypes such as resistance to hepatic steatosis and diet-induced obesity, and why long-time treatment with Compound B showed beneficial metabolic effects such as insulin-sensitization and reduction of the body weight gain and hepatic lipids.

Long-time treatment with Compound B for 4 weeks did not cause any skin aberrations in DIO mice. Long-time treatment with Compound A, in contrast to that with Compound B, caused moderate skin aberrations derived from atrophy of the sebaceous gland which were characterized by decrease amount and size of sebaceous gland acini. The skin aberrations associated with Compound A were not injurious or inflammatory changes, and the aberrations recovered 2 weeks after the compound withdrawal. As these skin aberrations resemble the observations in DGAT1 null mice, depletion of essential lipids due to DGAT1 inhibition is likely to be responsible for the aberrations. The results from the present study and DGAT1 null mice demonstrate that congenital DGAT1 is involved in maintenance and/or development of the sebaceous gland, producing fur essential lipids. As both Compound A and B inhibited the triglyceride synthesis, the lack of skin aberrations in the DIO mice treated with Compound B seems to be due to its minimal distribution to the skin.

Clinical data [25], [26] indicate that profound gastrointestinal (GI) adverse events such as nausea, diarrhea, and vomiting appear likely related to DGAT1 inhibition/loss of function in the GI tract. Ideally, a DGAT1 inhibitor as an oral therapeutic agent should overcome those adverse events. In the present study, we cannot deny the possibility that the DGAT1 inhibitor with intestine-restricted distribution may have the same problems in human. Although adverse events would, at least partly, be caused by the inhibition of intestinal DGAT1, their precise mechanisms are unclear. Further clinical studies of intestine-targeted compounds would clarify the therapeutic window of this type of DGAT1 inhibitors.

In conclusion, this study has shown for the first time that the intestine-targeted delivery of the DGAT1 inhibitor improves postprandial hyperlipidemia, body adiposity, hepatic lipid accumulation and insulin resistance without skin aberrations. The presently-observed improvement of metabolic abnormalities is in agreement with other reported results of the studies using DGAT1 inhibitors with different chemical structures [9]–[11], [27]–[28] or DGAT1 null mice. Collectively, our results provide pharmacological support for the hypothesis that the intestine is the key tissue in which DGAT1 plays a role in promoting obesity and insulin resistance when exposed to high fat food.

## Supporting Information

Figure S1 **Effects of Compound B on the hepatic steatosis.** Hematoxylin and Eosin staining of liver obtained from the diet-induced-obese (DIO) mice treated with vehicle, 10 or 30 mg/kg Compound B for 4 weeks.
(PDF)
