# Supplementary figures and images for: Intestine-Targeted DGAT1 Inhibition Improves Obesity and Insulin Resistance without Skin Aberrations in Mice
Source: PLoS One. 2014 Nov 18;9(11):e112027. doi: 10.1371/journal.pone.0112027 (PMC4236014; doi:10.1371/journal.pone.0112027)

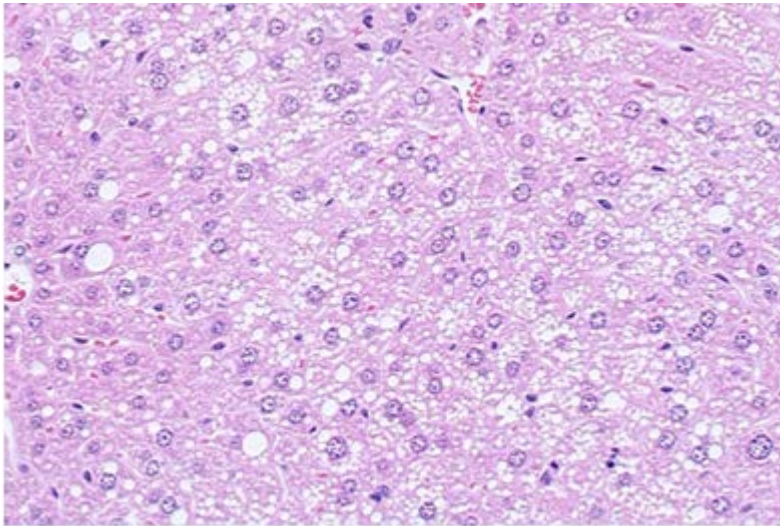

vehicle

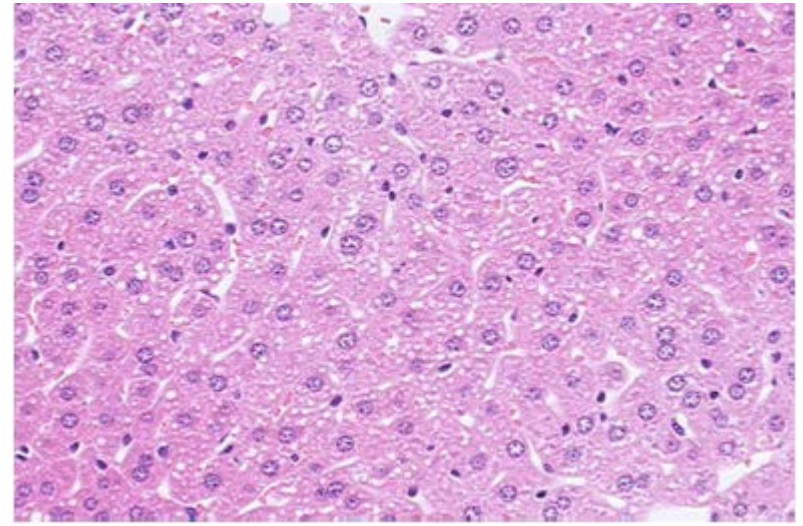

10 mg/kg

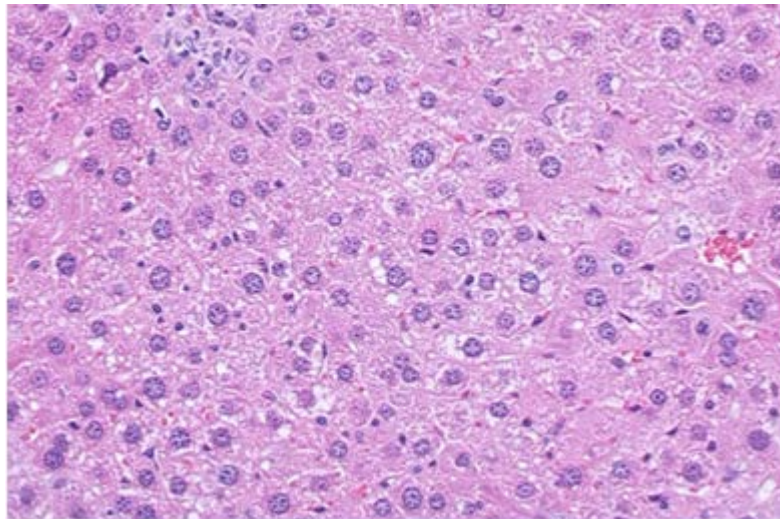

30 mg/kg

Supplement: Figure S1 — Effects of Compound B on the hepatic steatosis. Hematoxylin and Eosin staining of liver obtained from the diet-induced-obese (DIO) mice treated with vehicle, 10 or 30 mg/kg Compound B for 4 weeks. (PDF) [file pone.0112027.s001.pdf]
